# Development of a Visual Perception System on a Dual-Arm Mobile Robot for Human-Robot Interaction

**DOI:** 10.3390/s22239545

**Published:** 2022-12-06

**Authors:** Wei-Ting Weng, Han-Pang Huang, Yu-Lin Zhao, Chun-Yeon Lin

**Affiliations:** Department of Mechanical Engineering, National Taiwan University, Taipei 10617, Taiwan

**Keywords:** hand-gesture recognition, model-based tracking, visual-servo control, human-robot interaction, mobile robots

## Abstract

This paper presents the development of a visual-perception system on a dual-arm mobile robot for human-robot interaction. This visual system integrates three subsystems. Hand gesture recognition is utilized to trigger human-robot interaction. Engagement and intention of the participants are detected and quantified through a cognitive system. Visual servoing uses YOLO to identify the object to be tracked and hybrid, model-based tracking to follow the object’s geometry. The proposed visual-perception system is implemented in the developed dual-arm mobile robot, and experiments are conducted to validate the proposed method’s effects on human-robot interaction applications.

## 1. Introduction

Service robots that use human-robot interactions (HRIs) have been widely demonstrated to have superior performance with older adults, compared to those which use either human-machine interaction (HMI) or human-computer interaction (HCI) [1,2,3]. The elderly population is expected to reach two billion by 2050 [4]. Therefore, the potential demand for robots designed to care for the elderly and to keep them company is increasing daily. Robots are expected to significantly improve the quality of life of the elderly, while reducing the workload of human caregivers and addressing living and working needs [5]. In addition, robots can engage, interact, and communicate more naturally and effectively with human individuals under their care, thereby meeting social and entertainment needs. However, functionality and design of robots cannot be limited to a sociomedical perspective; a robot that falls short of the expectations and imaginations of elderly individuals may negatively affect their perceptions and experiences [6].

Typically, the elderly need to take specific medicines, drink fluids, maintain proper nutrition, and attend to other necessary daily routines. Yet, these behaviors increase their risk of falling and injury. At home, family members need constant attention and monitoring; in a care center, the constant need to perform small tasks for elderly residents can exhaust caregivers, especially when centers are often understaffed. Fortunately, robotics has the potential to serve as an alternative assistive solution for the long-term care of the elderly. A robot can use simultaneous localization and mapping (SLAM) to patrol within an environment and identify, from an image obtained through a camera, when an elderly individual needs help. Furthermore, by learning the needs of the elderly through dialogues, an appropriately equipped robot can use dual arms to track and grab required objects. Since robots have the potential to be used in many elderly-related areas, this paper focuses on using robots in general elderly care to reduce the workload of caregivers, improve the quality of life of the elderly, and maintain the normal daily activities of the elderly by integrating functions of hand-gesture recognition, visual feedback, and human-robot interaction into a dual-arm mobile robot.

In addition to language, humans use hand gestures to communicate. The shape of the hand established by the relative positions of fingers can represent different meanings. Hand-signaling systems can be divided into two types according to the method used to collect such data. The first employs a wearable device that uses various sensors to obtain information about the joints of the fingers and the palm. For example, soft-curvature sensor [7] changes resistance as it deforms under the pressure exerted on its embedded microchannel, and flexible-optical-fiber transducers measure angular displacements [8]. However, this method faces many challenges, such as hardware integration, short battery life, mechanical wear, and the need for a communication network. The second method uses non-contact sensors, such as cameras [9,10], radar sensors [11], and infrared (IR) detectors [12], to obtain the required information. For example, skeleton-based recognition [9] typically uses machine learning to obtain skeleton points of the hand; depth-based recognition [10] uses time-of-flight to obtain the depth of each pixel and distinguish the desired object through different depth intervals. It is worth mentioning that the hand’s natural warmth makes it a viable infrared light source for non-contact, human-machine interaction. The computer converts IR images into machine-readable signals, which can be recognized in low light or even darkness [12]. However, such systems are currently unable to identify more complex gestures. In conclusion, it is clear that the camera is not only one of the most ubiquitous sensors available for gesture recognition, but its accuracy and frequency also meet the needs of real-time use.

A visual servoing system combines robot vision and control [13], and its research fields encompass image processing, image detection, image tracking, robot kinematics and dynamics, and more. According to the different cues used for control, visual servoing can be divided into three categories [14,15,16]: One is position-based visual servoing (PBVS), the second is image-based visual servoing (IBVS), and the third is hybrid-based visual servoing (HBVS), also known as 2.5D visual servoing. In this framework, position-based visual servoing uses a Cartesian-pose-error signal to drive the robot to its goal. The error is obtained by comparing the current 3D pose with the desired views of an object. The current 3D pose, including position and orientation, and the desired 3D pose, which acts as the target, are reconstructed digitally using information extracted from features in the image. However, this method is sensitive to camera parameters due to its reliance on 3D reconstruction. Therefore, inaccurate camera parameters may cause many errors.

In contrast, the second category, image-based visual servoing, uses the error value of pixel-coordinate features as the input to drive the robot until the error is zero. Its unique strength lies in obtaining the error directly from the image space, making it remarkably robust in the face of errors in calibration and image noise, unlike calculations in a Cartesian space. A robot using an image-based system keeps moving to bring the current image features to the desired values. However, calculating the transformation from the error of feature points in pixel coordinates and translating this into commands to the robot are challenging. When the displacement value of pixel-coordinate features becomes too large, the camera may reach a local minimum or cross a singularity of the interaction matrix. Furthermore, the camera’s motion can become unpredictable due to the coupling of the related interaction matrix.

The third category, hybrid-based visual servoing, unites PBVS and IBVS methods, combining 2D and 3D information to direct a robot. It divides the tracking control of the object’s pose into two parts: The camera position is controlled directly in the image space, and the camera orientation is controlled by using motion estimation in a Cartesian space. One important consideration is how best to capture the necessary object information in the camera image because visual servoing relies on the information in the image to control a robot. Objects can be represented by points, primitive geometric shapes, silhouettes, and contours. Selecting the right features to detect an object is critical in tracking. The most commonly selected visual features are colors, textures, shapes, and spatial relationships. The four most common methods to detect these features are point detection, background subtraction, segmentation, and supervised learning. Point detectors include Harris, KLT, SIFT, SURF, FAST, and ORB. The position of an object relative to the scene can be acquired using two strategies: one uses markers, and the other uses markerless methods. In the first category, the end-effector adds a physical marker to calculate the transformation relationship between the end-effector and the camera [17]. The literature [18] shows one solution consisting of four main parts for markerless-visual servoing on unknown objects: treating it as a least-squares minimization problem, employing a recursive-Bayesian-filtering technique, treating it as a nonlinearly constrained optimization problem, and employing an image-based, visual-servo control. In the second category, the superquadric model method can be used to achieve markerless visual tracking. When the object to be tracked and its geometry are not complicated, Computer-Aided Design (CAD) model of the object is utilized for visual servoing, deploying real-time virtual visual servoing (VVS) to track and update the position between the object and the camera. In addition, You Only Look Once (YOLO) can be employed to extract the range of the CAD-bounding box to improve the effectiveness of CAD-based visual servoing [19].

Human behavior can be divided into explicit and implicit behavior, both essential to generating behavioral cues for a robot. The cues of explicit behavior, including speech, body language, sign language, vocalizations, facial expressions, actions, and the direction of gaze [20], all provide direct and intuitive messages in interactions. The cues of implicit behavior, including emotions, intention, social space, and personality, are covert since the information from others cannot be obtained directly; it must be inferred. Because behavioral cues and social signals differ less than individuals and cultures, only a small set needs to be recognized. Nevertheless, quantifying and measuring these cues and signals are challenging yet essential to successful HRI.

To measure the interaction between robots and humans in a more standardized way, the degree of interaction may be divided into four levels [21]: safety, comfort, naturalness, and sociability. Safety covers the minimum requirement when designing a robot; comfort ensures that interaction with a robot does not cause annoyance and pressure; naturalness is an advanced condition that captures the degree to which a robot acts like a human being, thereby achieving readability. Sociability reflects a robot’s ability to comply with high-level cultural conventions. Robots can make their judgments and comply with social norms and expectations. Historically, HRI research is divided into two approaches [22]: user-centric HRI and robot-centric HRI. Initially, pioneering robot engineers were understandably more focused on the growing capabilities of their creations; hence, HRI was robot-centric. Increasingly, robot designers have been taking a more holistic viewpoint, prioritizing the acquisition and development of specialized knowledge about how users perceive and regard products. This new user-centric HRI aims to accomplish human goals by delivering a high-quality user experience of robots that can perceive, cognize, and deal effectively with their surroundings.

Service robots mainly perform tasks targeting the well-being of humans in a semi- or fully autonomous way, unlike those targeting industrial-automation applications. Within this human-centered domain, multiple application areas exist, such as medical-robot assist, transportation robots, maintenance robots, shopping robots, and care robots [23,24]. Due to advances in medicine and healthcare, human lifespan has been considerably extended, bringing about a growing demand for healthcare services. Thus, the number of caregivers needed will increase day-by-day. Many of the problems caused by this rise of the elderly population can be averted by the application of service robots to elderly care. Care robots mainly provide physical, cognitive, or emotional support, including the completion of daily tasks, monitoring of behavior and health, and the provision of companionship [25,26,27,28]. To ensure quality of life, elderly individuals require respect, love, and help [29]. Therefore, for robots to fill this niche successfully, they need specific knowledge of how to care for the elderly and general recognition of human needs. 

Motivated by the need to develop a visual-servo system on a mobile-robot system, this paper illustrates a method that integrates the hand-gesture-recognition model, cognitive system, and virtual servoing into a developed, dual-arm mobile robot for human-robot interaction.

The remainder of this paper offers the following:

The visual-perception system on a mobile robot involves three subsystems. Hand-gesture recognition triggers an event where an elderly individual needs assistance. The cognitive system identifies the participation of the users. Visual servoing controls the dual-arm mobile robot to accomplish the tasks from the visual information. The visual-perception system is implemented on a developed, dual-arm mobile robot, and an example illustrates the applications of the visual system in human-robot interaction.

## 2. Visual Perception on a Dual-Arm Mobile Robot Design

**Figure 1:**
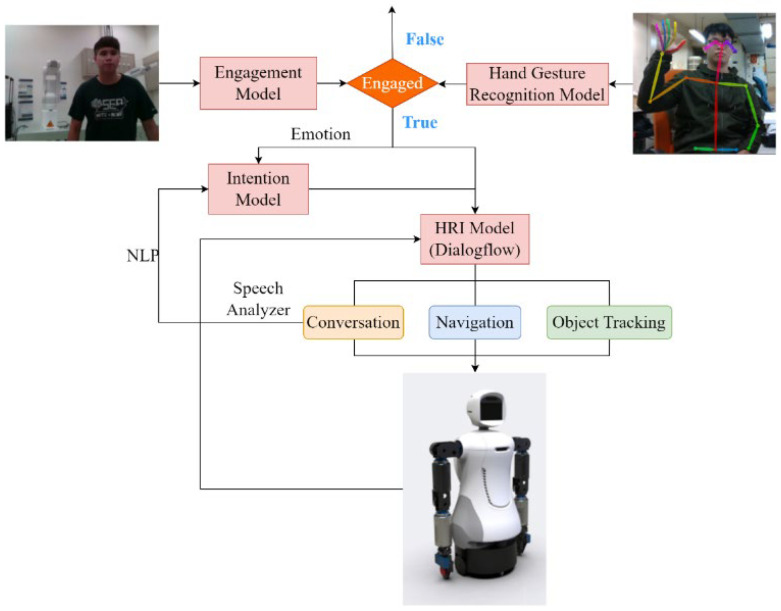
The robot’s cognitive system.

Figure 1 illustrates the architecture of the cognitive system of a dual-arm mobile robot, which utilizes the hand-gesture-recognition model to trigger HRI based on engagement, intention, and HRI models. In addition, visual tracking and an arm application are added to the HRI. 

### 2.1. Hand Gesture Recognition Model 

Many human behaviors can trigger interactions between humans and robots, one of which is gestures. The use of gestures is supported by 2D-pose estimation, a popular research topic that covers a wide range of applications. When an elderly individual has mobility problems or needs help, hand-gesture recognition is an intuitive way to alert the robot. The procedure for hand-gesture cognition includes extracting human-joint-point information, calculating feature vector, and obtaining intended gestures through feature comparison and voting. Score threshold of the required help is used to determine whether help is needed.

1. Feature Extraction

An open-source library, OpenPose [30,31], is utilized to obtain the positions of key points in the image space and their reliability.

2. Feature Matching

First, the feature vector of hand gesture for recognition is defined;the feature vector of test gesture and the feature vector of a pre-defined gesture is compared. If the test and pre-defined feature vectors are the same, the similarity is +1. If different, the similarity is −1. Similarity is unchanged if a zero-feature is in the test feature vector. Thus, the similarity between the test feature vector and a defined feature vector is between −14 and +14. Lastly, a similarity threshold is set if no similarity is higher than this threshold. The result of the recognition is an undefined hand gesture. The defined hand gesture with the highest similarity above the threshold is chosen as the result of the recognition, and the index of the defined hand gesture is returned.

3. Voting and Score

**Figure 2:**
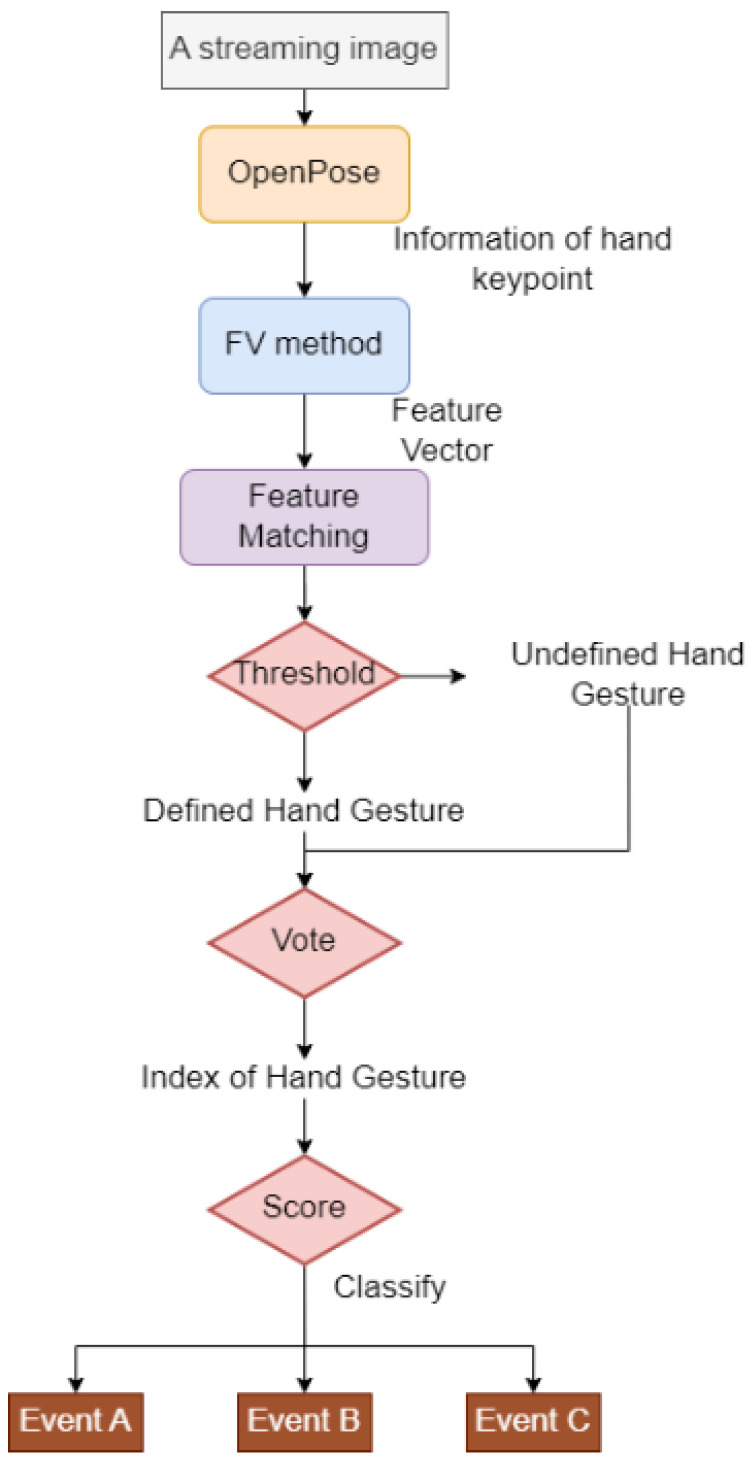
Schematic diagram of feature matching.

A single-frame image from the stream of images may not accurately represent the intended human gesture. It may just be a meaningless and temporary state, or a transitional state between two actions. Voting is utilized to filter data to eliminate any incorrect answer from a single recognition by OpenPose. Once a pre-defined list is populated with voted members, the program outputs the resulting gesture with the highest vote. However, the same static hand gesture may have different meanings in different situations. Scores based on different requirements are utilized to determine whether to trigger an event. The schematic diagram of the event trigger is shown in Figure 2. 

In our application, our primary objective is to use hand-gesture recognition to trigger an event in which an elderly individual needs help. Therefore, the gestures to initiate a help call must be simple and intuitive to the user. Because individuals in a variety of settings generally raise their hands to indicate that they need help, the necessary condition of the hand higher than the shoulder is utilized as the trigger gesture.

### 2.2. Cognitive System 

During an HRI, a robot must be able to identify when humans want to interact with it. Therefore, an engagement model is used to distinguish the level of engagement. The intention model judges the human individual’s intention by indirect information after an interaction is established. The HRI model is responsible for communication between the two participants in the interaction.

1. Engagement Model

**Figure 3:**
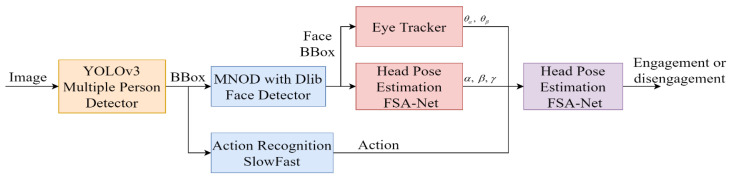
The architecture of the engagement model.

According to [32], engagement is a process by which individuals in an interaction start, maintain, and end their perceived connection with one another. Thus, engagement consists of four stages: the initial point of engagement, duration of engagement, disengagement, and reengagement. The laboratory model obtains the information required to build an engagement model through three information signals and a hidden Markov model (HMM); the architecture of this engagement model is shown in Figure 3 [33].

- Head Pose

Because eye gaze alone is not a reliable indicator, head pose is used to provide an approximation of eye gaze for attention recognition. Individuals are first detected using YOLOv3. The face detector of the OpenCV library is then used, along with a maximum margin target detector (MMOD); the orientation is finally obtained through a landmark-free method, FSA-Net [34]. Furthermore, yaw (*α*), pitch (*β*), and roll (*γ*) angles are used to distinguish whether individuals are facing the camera, as shown in the following equation:

(1) $$f(\alpha ,\;\beta ,\;\gamma )\{\begin{array}{cc} 1, & \left\|\alpha \right\|\leq \alpha^{\prime }\cap \left\|\beta \right\|\leq \beta^{\prime }\cap \left\|\gamma \right\|\leq \gamma^{\prime }\\ 0, & else \end{array}$$

where $\alpha^{\prime },\beta^{\prime },\gamma^{\prime }$ are the threshold of yaw, pitch, and roll, respectively.

- Eye Gaze

The image identified by the face detector is input to the gaze tracker, which is composed of a convolutional neural network (CNN). The horizontal and vertical angles of the eyes, produced as the output by the model, are used to check whether the agent is looking at the robot with the following equation:

(2) $$g\left(\theta_{\alpha },\;\theta_{\beta }\right)=\{\begin{array}{cc} 1, & \left\|\theta_{\alpha }\right\|\leq \theta_{\alpha }^{\prime }\cap \left\|\theta_{\beta }\right\|\leq \theta_{\beta }^{\prime }\\ 0, & else \end{array}$$

where $\theta_{\alpha }$, $\theta_{\beta }$ are the horizontal and vertical angles. $\theta_{\alpha }^{\prime }$, $\theta_{\beta }^{\prime }$ are their upper bounds.

- Action

Because motion recognition must consider human motion and the surrounding environment at the same time [35], it needs to operate at close to 30 frames per second (fps) in real-time applications. However, there are only a few key points in the stream. Therefore, the laboratory model uses a Two-Stream 3D-ConvNet and SlowFast [36] to obtain good efficiency and accuracy. The Kinetics-400 [37] dataset is used as the training data and is divided into interactions with a human and no interaction with a human, as shown in the following equation:

(3) $$h\left(a\right)=\{\begin{array}{cc} 1, & interaction\\ 0, & \mathrm{non-interaction} \end{array}$$

2. Intention Model

**Figure 4:**
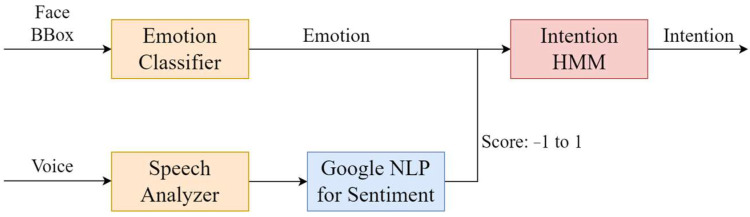
The architecture of the intention model.

Intention [38] is in a decision maker’s mind, so it cannot be directly observed or measured. Therefore, one must use the emotions of the interactor to infer intentions. To build an intention model, the laboratory model obtains intention through two information signals with three sentiment indexes and an HMM; the architecture of the intention model is shown in Figure 4 [33].

- Emotion Classifier

The laboratory uses a convolutional neural network (CNN) to construct an emotion classifier and uses FER-2013 [39] as the training dataset. The classifier is divided into seven emotions and their scores, which range from −1 (negative) to 1 (positive), among the three sentiment indexes, as shown in Table 1.

- Google NLP Sentiment

In addition to analyzing emotions through facial expressions, human emotions can be learned through language. The robot uses its microphone to obtain a recording of an individual’s voice and inputs it to the speech analyzer, Google Speech-to-Text API, and obtains the words with maximum likelihood, which are then input into Google ’s Natural Language Processing (NLP) sentiment analysis to obtain emotional scores and magnitudes. The sentiment is quantified and normalized, classified as positive, negative, or neutral, with a threshold of ±0.25.

3. Human-Robot Interaction Model

**Figure 5:**
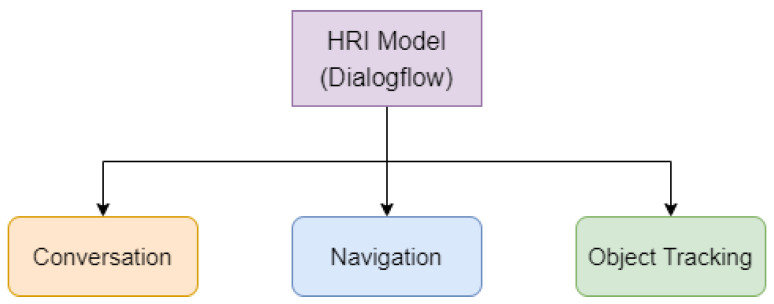
Score range of sentence sentiment.

**Figure 6:**
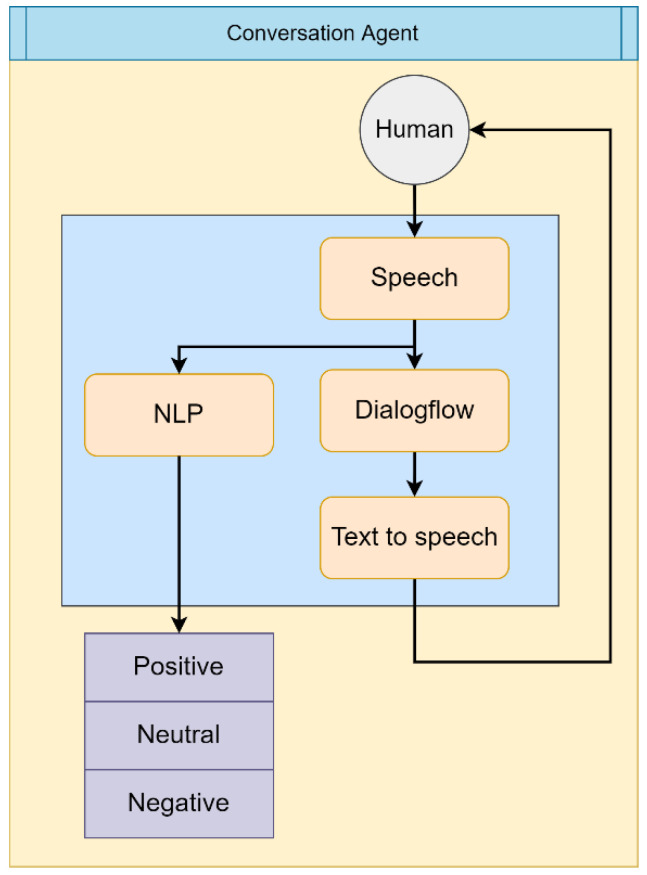
Schematic diagram of event trigger.

Service robots exist to aid humans and improve their quality of life. Accordingly, an HRI model establishes the communication necessary to assist. The interaction between robots and humans is carried out using Google Dialogflow as the core, so that the system can undertake a broader range of actions to understand the needs of an individual. Thus, HRI includes conversation, navigation, and object-tracking modules. The architecture of the HRI model with Google Dialogflow is shown in Figure 5. The flow chart of the related conversation is shown in Figure 6. 

### 2.3. Visual Servoing

Visual-servo control is a way of using image information to drive a robot. The difference between the desired and current feature points generates a velocity command to move a robot to the desired position. The greater the error, the greater the velocity; conversely, as the error decreases, so does the velocity. The error decreases to within a tolerable range as an exponential function. Because hybrid, model-based tracking only uses an object’s geometry for tracking, it cannot recognize what the object is. Therefore, YOLOv4 is used to confirm whether an object is the intended target before tracking.

1. YOLOv4

YOLOv4 obtains an object’s centroid position, the bounding box’s length and width, and the probability of a match. The obtained ID has a corresponding category, confirming whether an object is the one to be tracked.

2. Hybrid Model-based Tracking (HMBT)

**Figure 7:**
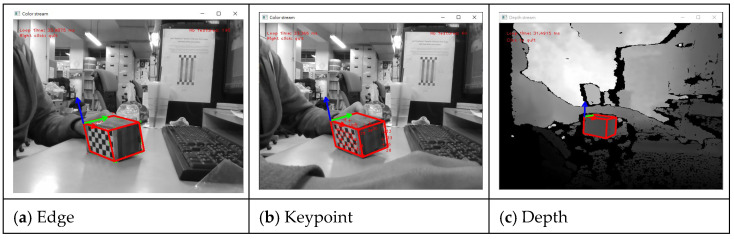
Display of the three features for tracking. (**a**) The edge features. (**b**) The keypoint features. (**c**) The depth features.

**Figure 8:**
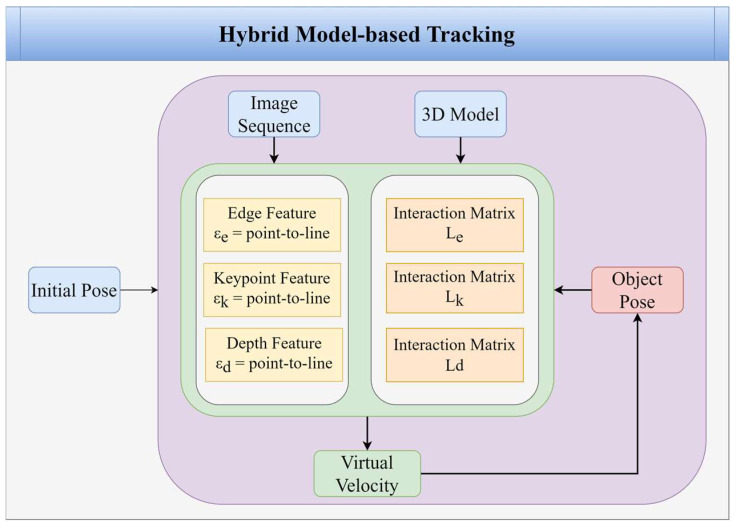
The architecture of the hybrid model-based tracking.

Hybrid model-based tracking is employed to achieve more robust tracking by tracking the edges of the model, keypoint features, and depth surfaces. In this scheme, moving edges and the color camera handle the depth sensor’s keypoint and normal depth features, as shown in Figure 7. The architecture of the proposed hybrid model-based tracking is shown in Figure 8.

3. Virtual Visual Servoing (VVS)

The pose estimation of object coordinates is an intermediate step that uses the image for control. Pose computation is obtained through 3D-coordinate points (or other geometric features, such as lines, circles) and their 2D projections onto the image plane. Virtual visual servoing (VVS), similar to 2D visual servoing [40], is a numerical method for full-scale, nonlinear optimization. The approach estimates the object pose by minimizing the error Δ between the desired state s* and the current state *s*, with the error *e* decreasing exponentially, as shown in the following:

(4) $$e=(s(r)-s^{*})$$

(5) $$\dot{e}=-\lambda e$$

where *r* is the estimated pose, and *λ* is the positive scalar.

The interaction matrix is then used to link the error change $\dot{e}$ and the virtual camera velocity *v* as follows:

(6) $$\dot{e}=L_{s}v$$

where *L_s_* is the interaction matrix that depends on the value of image features *s* and their corresponding depth *Z* in the scene. From Equations (4) and (5), Equation (6) is obtained, and the virtual-camera velocity *v* is obtained by comparing the features of the desired state s* with those of the current state *s* at each iteration:

(7) $$v=-\lambda {L^{+}}_{s}(s(r)-s^{*})$$

where ${L^{+}}_{s}$ is the pseudoinverse of $L_{s}\in {\mathbb{R}}^{2n\times 6}$, $v\in {\mathbb{R}}^{6\times 1}$, $s(r)-s^{*}\in {\mathbb{R}}^{2n\times 1}$, and *n* is the number of the feature points.

Therefore, the pose of the *k*th iteration can be updated by Equation (7). Δ*T* is the transformation between the *k*th and (*k* + 1)th iterations, in which a six-dimensional vector is changed into a four-dimensional matrix through the ∧ operation, and an exponential map is created as in the following:

(8) Toc(k+1)=ΔT−1Tock

(9) ΔT=Tc(k+1)ck=exp(v∧)

where $v=\left[\begin{array}{c} \left[\begin{array}{c} v_{x}\\ v_{y}\\ v_{z} \end{array}\right]\\ \left[\begin{array}{c} w_{x}\\ w_{y}\\ w_{z} \end{array}\right] \end{array}\right]=\left[\begin{array}{c} \rho_{3\times 1}\\ \phi_{3\times 1} \end{array}\right]$; $v^{\wedge }=\left[\begin{array}{cc} \phi^{\wedge } & \rho \\ 0_{1\times 3} & 0_{1\times 1} \end{array}\right]$; $\phi^{\wedge }=\left[\begin{array}{ccc} 0 & -\omega_{z} & \omega_{y}\\ \omega_{z} & 0 & -\omega_{x}\\ -\omega_{y} & \omega_{x} & 0 \end{array}\right]$ $\Delta T\in {\mathbb{R}}^{4\times 4}$, $v\in {\mathbb{R}}^{6\times 1}$, $v^{\wedge }\in {\mathbb{R}}^{4\times 4}$.

**Figure 9:**
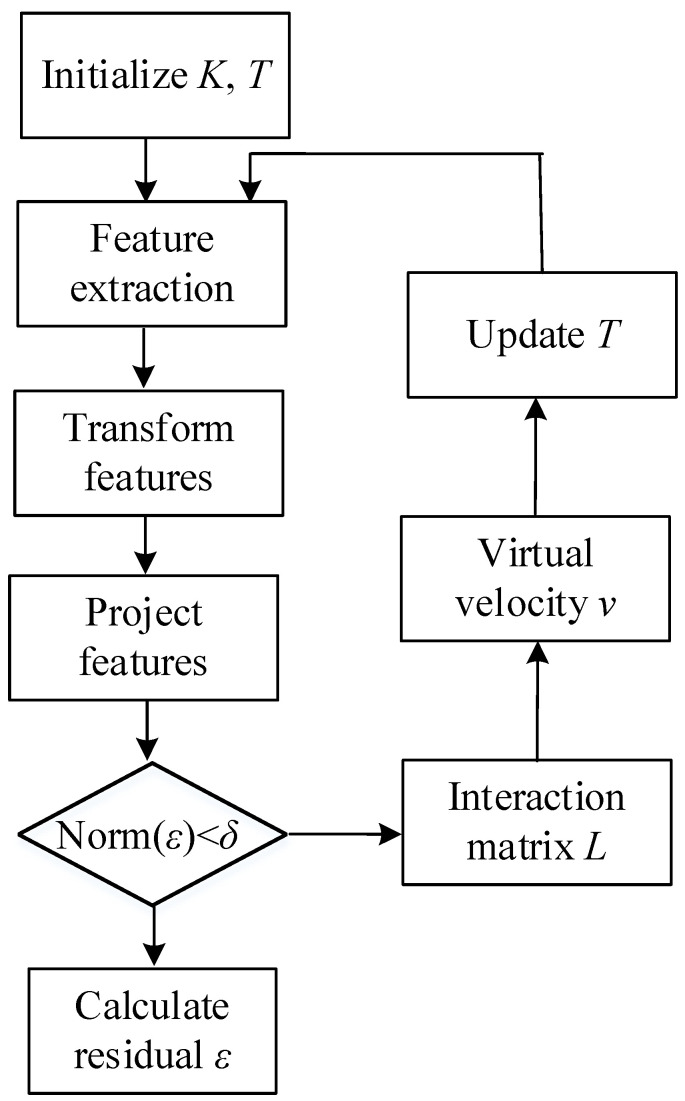
Algorithm of the VVS method.

At each iteration, virtual velocity *v* updates the transformation matrix *T* until the error between the current and desired features is less than the threshold. Thus, the correct pose between the camera and the object can finally be obtained. The pseudocode and flowchart are shown in Algorithm 1 and Figure 9, respectively. 

**Algorithm** **1** Pseudocode of the VVS algorithm.Initialize(); // get camera’s model parameters K and initial pose T $\mathrm{Get}\;\mathrm{the}\;\mathrm{desired}\;\mathrm{features}\;s^{*}$;While (true) {        Transform the model from object frame to camera frame by T;        Project the model to image plane by K;        Extract the feature s;        $\varepsilon =s-s^{*}$; // get the error between the current and desired feature        if (norm(ε)<threshold) {                break;        }        else {                Calculate the interaction matrix L;                $v=-\lambda L^{+}\varepsilon$; // get the virtual velocity                 T=Toc(k+1)=exp(v^)−1⋅ckTo; // update T        }}$T^{*}=T$;

The mobile-robot system is divided into non-platform and platform parts. The non-platform part has two arms as two end-effectors, which use six variables to represent the position and orientation of each end-effector, and eighteen joints as active frames, as shown in Equations (10a) and (10b). The other is the mobile platform, a non-holonomic constraint created in Equations (11a) and (11b).

(10a) $$p_{a}={\left[{\dot{p}}_{1}\;{\dot{p}}_{2}\right]}^{T}\in {\mathbb{R}}^{12\times 1},q_{a}={\left[{\dot{q}}_{1}\;{\dot{q}}_{2}\;\cdots \;{\dot{q}}_{18}\right]}^{T}\in {\mathbb{R}}^{18\times 1}$$

(10b) $${\dot{p}}_{a}=J_{a}{\dot{q}}_{a}\;\Rightarrow \;\left[\begin{array}{c} {\dot{p}}_{1}\\ {\dot{p}}_{2} \end{array}\right]=\left[\begin{array}{ccc} \begin{array}{c} 0\\ 0 \end{array} & \begin{array}{c} J_{aR}\\ 0 \end{array} & \begin{array}{c} 0\\ J_{aL} \end{array} \end{array}\right]\left[\begin{array}{c} {\dot{q}}_{1}\\ {\dot{q}}_{2}\\ \vdots \\ {\dot{q}}_{18} \end{array}\right],\;{\dot{p}}_{a}\in {\mathbb{R}}^{12\times 1},\;{\dot{q}}_{a}\in {\mathbb{R}}^{18\times 1},\;J_{a}\in {\mathbb{R}}^{12\times 18}$$

(11a) $$p_{m}={\left[x\;y\;\theta_{z}\right]}^{T}\in {\mathbb{R}}^{3\times 1},q_{m}={\left[s\;\theta_{z}\right]}^{T}\in {\mathbb{R}}^{2\times 1}$$

(11b) $${\dot{p}}_{m}=J_{m}{\dot{q}}_{m}\;\Rightarrow \;\left[\begin{array}{c} \dot{x}\\ \begin{array}{c} \dot{y}\\ \begin{array}{c} \dot{z}\\ \begin{array}{c} {\dot{\theta }}_{x}\\ \begin{array}{c} {\dot{\theta }}_{y}\\ {\dot{\theta }}_{z} \end{array} \end{array} \end{array} \end{array} \end{array}\right]=\left[\begin{array}{cc} \begin{array}{c} \cos\left(\theta \right)\\ \begin{array}{c} \sin\left(\theta \right)\\ \begin{array}{c} 0\\ \begin{array}{c} 0\\ \begin{array}{c} 0\\ 0 \end{array} \end{array} \end{array} \end{array} \end{array} & \begin{array}{c} 0\\ \begin{array}{c} 0\\ \begin{array}{c} 0\\ \begin{array}{c} 0\\ \begin{array}{c} 0\\ 1 \end{array} \end{array} \end{array} \end{array} \end{array} \end{array}\right]\left[\begin{array}{c} v\\ w \end{array}\right],\;{\dot{p}}_{m}\in {\mathbb{R}}^{6\times 1},\;{\dot{q}}_{m}\in {\mathbb{R}}^{2\times 1},\;J_{m}\in {\mathbb{R}}^{6\times 2}$$

4. Mobile Platform Motion Strategy

**Figure 10:**
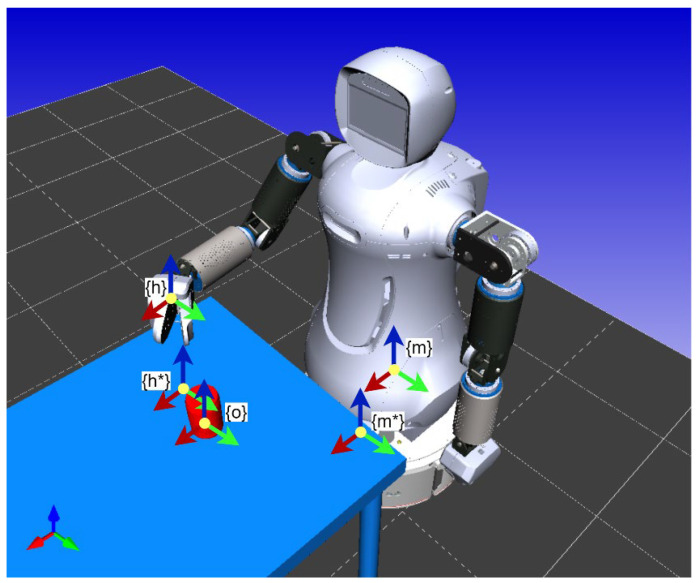
Frames definition for visual-servo control. The symbol (*) is used to represent the desired positions.

When a robot is in a state far from the target object, the motion of the robot is driven by the error between the desired position of the robot base and its current position. Odometry is utilized to obtain the current pose of the robot base to determine the robot’s location in this environment. Hybrid, model-based tracking and coordinate transformation can be used to calculate the desired position of the robot base. Once the target object is calculated to be within grasping distance, the robot tracks the object until *m* and m* coincide in Figure 10 so that it stays inside the workspace of the arms. Thus, *s* is defined as the difference between the desired and the current positions of the mobile platform, based on task requirements.

(12) s=(tmm*, θu),s*=(0, 0)

The velocity of the mobile platform Vmm(∈ℝ6×1)=[x˙y˙z˙θ˙xθ˙yθ˙z]T is given as

(13a) Vmm6×1=−λLe+e(t)

(13b) Le=[Rmm*00Lθu]

(13c) e(t)=s−s*=(tmm*, θu)

where λ is a positive scalar. Inverse kinematics is used to calculate the linear and angular velocities of the mobile platform obtained by

(14) $${\dot{q}}_{m}=\left(\begin{array}{c} v\\ w \end{array}\right)={\left(\begin{array}{cc} \begin{array}{c} \cos\theta \\ \sin\theta \\ 0\\ \begin{array}{c} 0\\ 0\\ 0 \end{array} \end{array} & \begin{array}{c} 0\\ 0\\ 0\\ \begin{array}{c} 0\\ 0\\ 1 \end{array} \end{array} \end{array}\right)}^{-1}\left(\begin{array}{c} \dot{x}\\ \begin{array}{c} \dot{y}\\ \dot{z}\\ \begin{array}{c} {\dot{\theta }}_{x}\\ {\dot{\theta }}_{y}\\ {\dot{\theta }}_{z} \end{array} \end{array} \end{array}\right)=-\lambda {J_{m}}^{+}{L_{e}}^{+}e\left(t\right).$$

Moreover, tracking weight, which is added to adjust the tracking speed in the six-dimensional pose, is determined by

(15) $${\dot{q}}_{m}=\left(\begin{array}{c} v\\ w \end{array}\right)={\left(\begin{array}{cc} \begin{array}{c} \cos\theta \\ \sin\theta \\ 0\\ \begin{array}{c} 0\\ 0\\ 0 \end{array} \end{array} & \begin{array}{c} 0\\ 0\\ 0\\ \begin{array}{c} 0\\ 0\\ 1 \end{array} \end{array} \end{array}\right)}^{-1}\left(\begin{array}{cccc} \begin{array}{c} w_{1}\\ 0\\ 0\\ \begin{array}{c} 0\\ 0\\ 0 \end{array} \end{array} & \begin{array}{c} 0\\ w_{2}\\ 0\\ \begin{array}{c} 0\\ 0\\ 0 \end{array} \end{array} & \begin{array}{c} 0\\ 0\\ w_{3}\\ \begin{array}{c} 0\\ 0\\ 0 \end{array} \end{array} & \begin{array}{ccc} \begin{array}{c} 0\\ 0\\ 0\\ \begin{array}{c} w_{4}\\ 0\\ 0 \end{array} \end{array} & \begin{array}{c} 0\\ 0\\ 0\\ \begin{array}{c} 0\\ w_{5}\\ 0 \end{array} \end{array} & \begin{array}{c} 0\\ 0\\ 0\\ \begin{array}{c} 0\\ 0\\ w_{6} \end{array} \end{array} \end{array} \end{array}\right)\left(\begin{array}{c} \dot{x}\\ \begin{array}{c} \dot{y}\\ \dot{z}\\ \begin{array}{c} {\dot{\theta }}_{x}\\ {\dot{\theta }}_{y}\\ {\dot{\theta }}_{z} \end{array} \end{array} \end{array}\right)=-\lambda {J_{m}}^{+}W_{m}{L_{e}}^{+}e\left(t\right)$$

The pseudocode of the visual-servo control is shown in Algorithm 2.

**Algorithm** **2** Pseudocode of the visual-servo control of the mobile platformWhile (true) {        Calculate s=(tmm*, θu)$,\;s^{*}=\left(0,\;0\right)$;        Calculate e=s−s*=(tmm*, θu);        if (|e(0)|<0.005 && |e(1)|<0.005) {                if (|e(5)|<0.01) {                        break;                }                else {                        $e(5)=s(5)-s^{*}(5)$;                        $\left(\begin{array}{c} v\\ w \end{array}\right)=-\lambda {J_{m}}^{+}W_{m}{L_{e}}^{+}e$;                        Cmd(v, w);                }        }        else {                $e(5)=Rz_{Mobi}-\tan^{-1}(e(1)/e(0))$;                $\left(\begin{array}{c} v\\ w \end{array}\right)=-\lambda {J_{m}}^{+}W_{m}{L_{e}}^{+}e$                Cmd(v, w);        }}

5. Arm Motion Strategy

When the robot is inside its workspace, its main task is to accurately grasp the target object. In this step, the robot tracks the target object until *h* and h* coincide, as shown in Figure 10, so that the end-effector can grasp it. Thus, *s* is defined as the difference between the desired and the current poses of the end-effector, based on task requirements.

(16) s=(thh*, θu),s*=(0, 0)

The velocity of the mobile platform Vhh(∈ℝ6×1)=[x˙y˙z˙θ˙xθ˙yθ˙z]T is given as

(17a) Vhh6×1=−λLe+e(t)

(17b) Lh=[Rhh*00Lθu]

(17c) e(t)=s−s*=(thh*, θu)

The velocity of the end-effector ${\dot{q}}_{a}$ can be obtained by

(18) q˙=Ja+Vhh=−λJa+Le+e(t)

Inverse kinematics is used to calculate the angular velocity of the motors

(19) $$\dot{q}={J_{a}}^{+}\left(\begin{array}{cccc} \begin{array}{c} w_{1}\\ 0\\ 0\\ \begin{array}{c} 0\\ 0\\ 0 \end{array} \end{array} & \begin{array}{c} 0\\ w_{2}\\ 0\\ \begin{array}{c} 0\\ 0\\ 0 \end{array} \end{array} & \begin{array}{c} 0\\ 0\\ w_{3}\\ \begin{array}{c} 0\\ 0\\ 0 \end{array} \end{array} & \begin{array}{ccc} \begin{array}{c} 0\\ 0\\ 0\\ \begin{array}{c} w_{4}\\ 0\\ 0 \end{array} \end{array} & \begin{array}{c} 0\\ 0\\ 0\\ \begin{array}{c} 0\\ w_{5}\\ 0 \end{array} \end{array} & \begin{array}{c} 0\\ 0\\ 0\\ \begin{array}{c} 0\\ 0\\ w_{6} \end{array} \end{array} \end{array} \end{array}\right)\left(\begin{array}{c} \dot{x}\\ \begin{array}{c} \dot{y}\\ \dot{z}\\ \begin{array}{c} {\dot{\theta }}_{x}\\ {\dot{\theta }}_{y}\\ {\dot{\theta }}_{z} \end{array} \end{array} \end{array}\right)=-\lambda {J_{a}}^{+}W_{a}{L_{e}}^{+}e\left(t\right)$$

where $W=diag(\begin{array}{cccccc} w_{1} & w_{2} & w_{3} & w_{4} & w_{5} & w_{6} \end{array})$.

**Figure 11:**
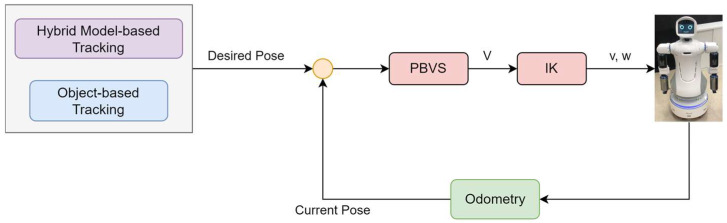
Block diagram of the visual-servo control for the arms.

Moreover, tracking weight is added to adjust the tracking speed in the six-dimensional pose, as shown in Equation (19). Figure 11 shows the block diagram of the visual-servo control for the dual arms, and the pseudocode of the visual-servo control applied on the dual arms is shown in Algorithm 3. 

**Algorithm** **3** Pseudocode of the visual-servo control of the robotic armsWhile (true) {        Calculate s=(thh*, θu)$,\;s^{*}=\left(0,\;0\right)$;        Calculate e=s−s*=(thh*, θu);        if (|e(3)|<0.02 && |e(4)|<0.02 && |e(5)|<0.02) {                if (|e(0)|<0.01 && |e(1)|<0.01 && |e(2)|<0.01) {                        break;                }                else {                        $\left(\begin{array}{c} v\\ w \end{array}\right)=-\lambda {J_{a}}^{+}W_{a}{L_{e}}^{+}e$                        $p^{\prime }=p+\left(\begin{array}{c} v\\ w \end{array}\right)$                         $q=IK(p^{\prime })$                         Cmd(q)                }        }        else {                $\left(\begin{array}{c} v\\ w \end{array}\right)=-\lambda {J_{a}}^{+}W_{a}{L_{e}}^{+}e$                $p^{\prime }=p+\left(\begin{array}{c} v\\ w \end{array}\right)$                Cmd(q);        }}

## 3. Simulation and Experiment Results

**Figure 12:**
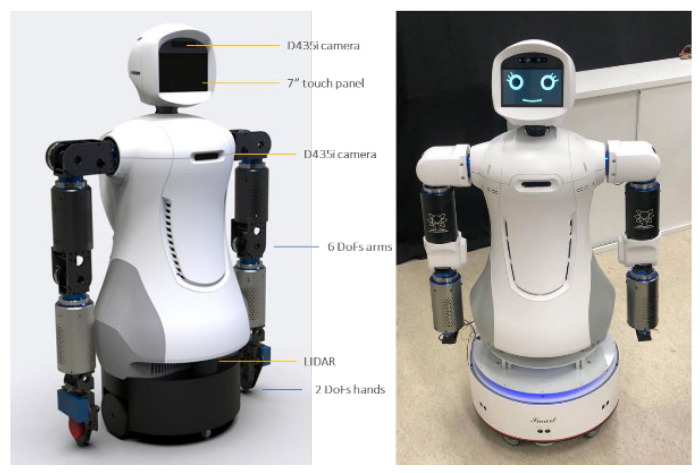
The mobile robot Mobi.

**Figure 13:**
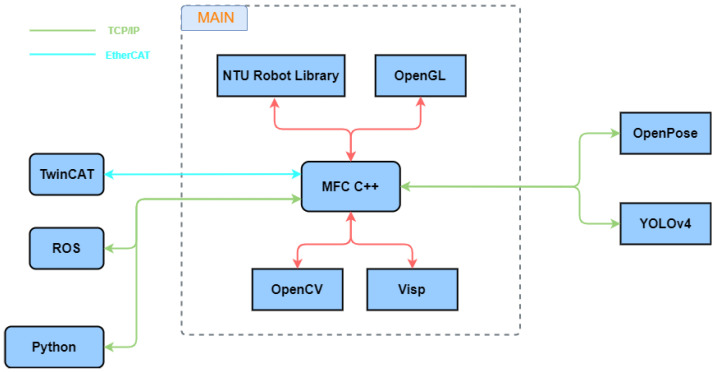
Soft architecture.

The laboratory developed a mobile robot named Mobi, shown in Figure 12. The robot system mainly consists of three computers—Win10 computer, NVIDIA Jetson AGX Xavier, and Linux industrial personal computer. We run most of the programs in an Intel Core i7-9700k desktop computer with NVIDIA GeForce GTX 1650 GPU and 32Gb RAM, which controls dual arms and hands, and communicates with other computers. The two RealSense D435i cameras are connected to the Win10 computer; due to the different positions, they are used to identify the user’s expression and observe the grasping situation. NVIDIA Jetson AGX Xavier is famous for its robust GPU computation for deep learning. Therefore, it is used to process real-time predictions from raw images. Linux industrial personal computer controls robot movement and navigation based on the robot operating system (ROS). The software structure developed by our laboratory for the robot is shown in Figure 13.

**Figure 14:**
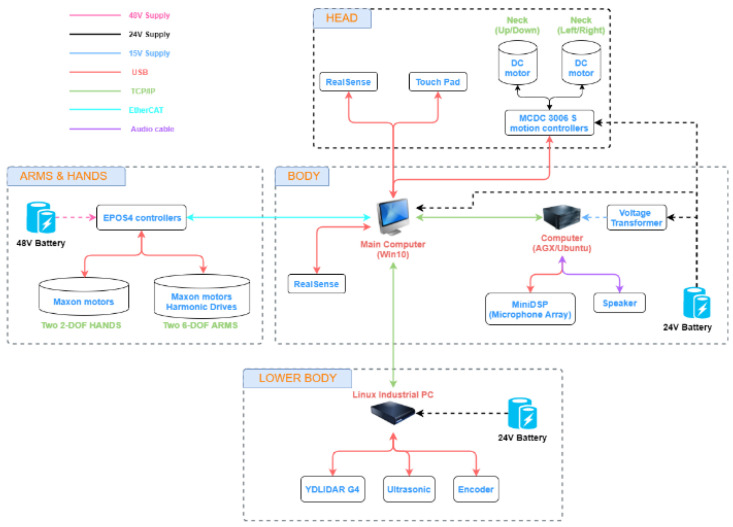
The hardware architecture of the mobile-robot system.

The hardware architecture of Mobi is demonstrated in Figure 14. The intention is that Mobi will be able to understand elderly users using hand-gesture recognition, the engagement and intention models, and the conversation system. A dual-arm system and HMBT with YOLOv4 will be used to grab the required objects to demonstrate the wide range of effective care a robot can provide.

**Figure 15:**
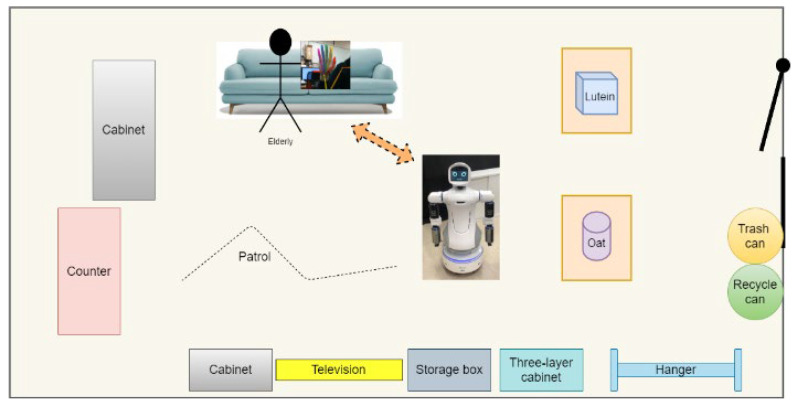
Experimental scenario of long-term care centers.

**Figure 16:**
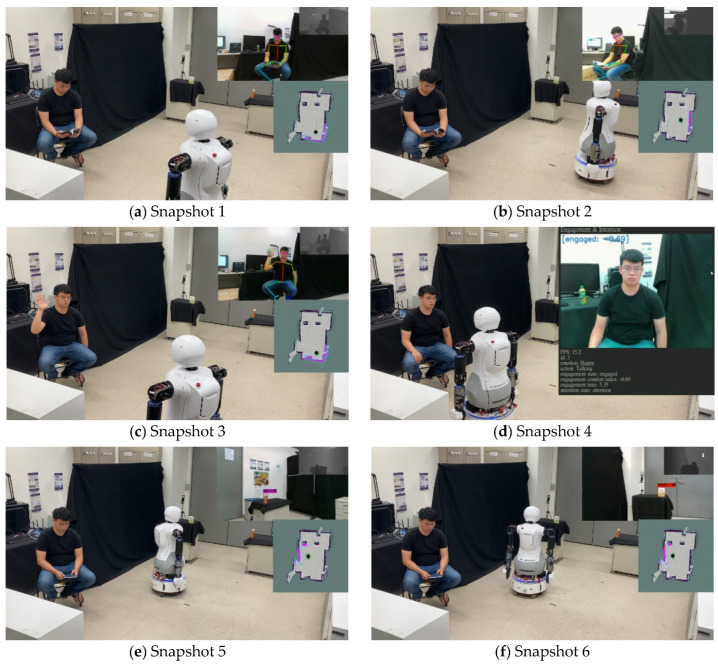
Snapshots from patrol to a conversation, then identifying the object to be tracked. (**a**,**b**) Patrol. (**c**) Recognition of a trigger gesture for human-robot interaction. (**d**) Deployment of engagement and intention model. (**e**,**f**) Identification of the object to be tracked.

**Figure 17:**
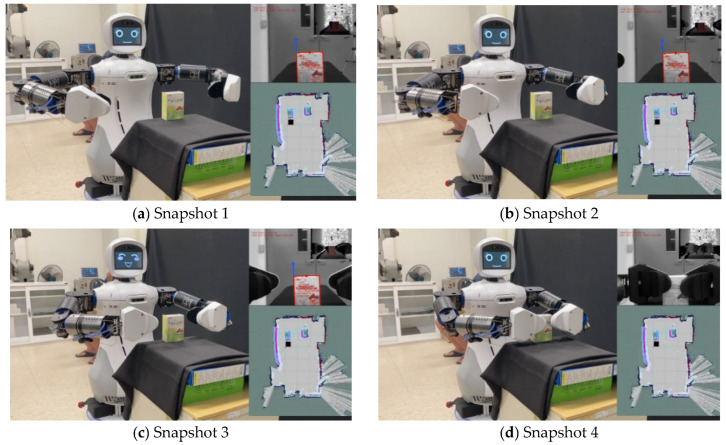
Snapshots of the dual-arm robot using a hybrid, model-based tracking method to track objects using its arms. (**a**–**d**) The process for the robotic grasping.

Figure 15 shows the experimental scenario, and the snapshots in Figure 16 show the transitions in Mobi’s behavior from patrol to a conversation, and finally, to identify an object to be tracked. First, OpenPose is used to extract skeleton information of the human body. The human individual’s hand gesture then initiates hand-gesture recognition (c), which triggers HRI. Subsequently, a conversation system (d) composed of Google APIs is used to chat with the user to ascertain the exact needs of the human subject. Finally, YOLO is used to identify the required object using bounding boxes. Subsequently, Mobi is able to use the hybrid, model-based tracking method to track and transport the target object. Figure 17 shows the dual arms grasping an object.

## 4. Conclusions

The visual-perception system of a dual-arm mobile robot for human-robot interaction is presented. The proposed visual-servoing system integrates multiple subsystems and applications, including hand-gesture recognition, a visual-servoing system, and the application of dual arms to the fundamental architecture of the cognitive system. The hand-gesture-recognition system uses feature vectors extracted through OpenPose to recognize one or two-handed human gestures. These can then be used to control the robot and trigger HRI. The visual-servoing system uses YOLOv4 to identify the object to be tracked. The system is further supported by hybrid, model-based tracking, which tracks the object’s geometry and oversees motion planning. Experiments demonstrate the various functions of the integrated system, and the results confirm the effectiveness of the proposed method.

Future work would include more scenarios and tests based on the user experience of elderly users to develop a more extensive range of applications of this sensing system. The user experience of elderly users will be considered to verify and improve the effectiveness of interventions by the robot system on the lives of senior adults.

## Figures and Tables

**Table 1:** Sentiment indexes of emotion.

	**Sentiment Index**		
	**Positive**	**Neutral**	**Negative**
Emotion	Happy (0.9)	Neutral (−0.1)	Disgust (−0.9)
			Angry (−0.9)
		Surprise (0.3)	Fear (−0.8)
			Sad (−0.9)

## Data Availability

Not applicable.
